# Childhood tuberculosis and treatment outcomes in Accra: a retrospective analysis

**DOI:** 10.1186/s12879-019-4392-6

**Published:** 2019-08-28

**Authors:** Sally-Ann Ohene, Sarah Fordah, Prince Dela Boni

**Affiliations:** 1 World Health Organization Country Office, Accra, Ghana; 2 Ghana Health Service, Kumasi, Ghana; 3 Ghana Institute of Management and Public Administration, Accra, Ghana

**Keywords:** Ghana, TB, Children, Treatment outcomes

## Abstract

**Background:**

Tuberculosis (TB) is a leading cause of death in children and adults. Unlike for adults, there is paucity of data on childhood TB in several countries in Africa. The study objective was to assess the characteristics and treatment outcomes of children with TB from multiple health facilities in Accra, Ghana.

**Methods:**

A retrospective analyses was conducted using secondary data on children less than 15 years collected from 11 facilities during a TB case finding initiative in Accra from June 2010 to December 2013. Demographic and clinical characteristics as well as treatment outcomes were assessed. Multivariable logistic regression was conducted to assess predictors of mortality.

**Results:**

Out of the total 3704 TB cases reported, 5.9% (219) consisted of children with a female: male ratio of 1:1.1. Children less than 5 years made up 56.2% of the patients while 44.2% were HIV positive. The distribution of TB type were as follows: smear positive pulmonary TB (SPPTB), 46.5%, clinically diagnosed pulmonary TB 36.4%.%, extra-pulmonary TB 17.4%. Among the 214 children (97.7%) for whom treatment outcome was documented, 194 (90.7%) were successfully treated consisting of 81.3% who completed treatment and 9.4% who were cured. Eighteen children (8.4%) died. Mortality was significantly higher among the 1–4 year group (*p* < 0.001), those with SPPTB (p < 0.001) and HIV positive children (p < 0.001). In logistic regression, SPPTB and HIV positivity were predictors of mortality.

**Conclusion:**

The proportion of children in Accra successfully treated for TB met the target of END TB Strategy treatment success indicator. HIV positivity was a risk factor for death. Reducing mortality in TB-HIV co-infected children will further improve treatment outcomes of children with TB in Accra.

## Background

It is estimated that there were 1million new cases of tuberculosis (TB) among children less than 15 years globally in 2017 representing 10% of the TB cases worldwide [1]. About 234,000 children were also projected to have died from TB in the same year [1]. This burden highlights the need to surmount the barriers to tackling TB in children which is identified as one of the top causes of death in this age group [2, 3]. There has been a growing interest in childhood TB with the recognition that efforts at TB control are bound to fail if children who serve as potential sources of future infection are ignored [2, 4]. This is in addition to the compelling obligation to diagnose and treat a disease for which drugs are available to effect cure [4]. Considering that TB in childhood also serves as a marker for recent disease transmission and therefore control activities, it is important for national TB control programs to have a good understanding of the burden of childhood TB in the respective settings [4]. This will facilitate the adoption of appropriate strategies to implement the END TB Strategy which among others call for the expansion of services to manage TB among children [5].

Over the years several studies have been carried out in African countries that have thrown light on the burden, diagnosis and treatment outcomes of childhood TB [6–11]. While some studies on TB in adults have been published in Ghana, there is very limited data on childhood TB to inform programming and planning for this age group [12–14]. To address this gap, this study was undertaken with the objective of assessing the demographic and clinical characteristics as well as treatment outcomes and risk factors for mortality among children diagnosed with TB from multiple health facilities in Accra, Ghana.

## Methods

### Study design, setting and population

The study was retrospective in nature analyzing secondary data from a database of TB patients diagnosed from June 2010 to December 2013 during a TB case finding initiative implemented in 11 health facilities in Accra, the capital of Ghana. The main strategies of the TB case finding initiative involved identification of patients suspected to have TB through symptomatic screening in the OPDs and HIV clinics in these facilities and contact tracing. The presumed TB patients identified through the screening process were investigated for TB and those confirmed to have TB were put on TB treatment as per the NTP guidelines. The 11 public health facilities participating in the initiative included a children’s hospital which provided in and outpatient care for children, 3 polyclinics which had only out-patient services for all ages and seven hospitals which consisted of 5 general hospitals, a regional hospital and a teaching hospital all providing both in and outpatient services. For the analyses the regional and teaching hospitals which are referral facilities providing more specialized care were grouped into one category termed “specialized” hospitals to distinguish them from the general hospitals. These facilities which implement the directly observed treatment short course (DOTS) strategy in line with the Ghana National TB Control Program (NTP) guidelines together manage about 70% of TB cases in Accra. Further details of the initiative are described elsewhere [15].

The study population consisted of children less than 15 years identified during the period of the case finding initiative over the stated period. Children below 15 years make up approximately 29% of Accra’s population [16]. The NTP guidelines for the diagnosis and management of TB in children made use of a screening tool adapted from Osborne’s scoring system. The tool revolved around the identification of clinical features including cough, weight loss or failure to gain weight and history of contact with a TB patient to detect children suspected to have TB [17–19]. In the event that the child was able to produce sputum or gastric lavage could be performed, 2 samples were sent for smear microscopy for acid fast bacilli. Although culture services were accessible, GeneXpert services were not readily available during the time of the initiative. Children in whom smear results were positive were classified as smear positive TB. Those with smear negative results were designated smear negative TB if after further evaluation by means of a thorough clinical assessment, chest X-ray, tuberculin skin test, the clinician determined the child had TB. Extra-pulmonary TB (EPTB) was also diagnosed after various relevant investigations had been conducted on samples obtained from children with suggestive clinical presentations. Examples of samples included cerebrospinal fluid for TB meningitis and fine needle aspiration of suspected TB adenitis [17, 18]. Children designated as having clinically diagnosed TB were those who may not have been able to produce sputum or for whom gastric lavage could not be done and so did not have sputum microscopy done but had physical signs, chest X-ray suggestive of pulmonary TB, a history of contact with a person infected with TB and were assessed by a clinician to have TB. Children diagnosed with TB were put on a standard 6 months regimen as per the NTP and WHO guidelines consisting of a 2-month intensive phase with 4 drugs (isoniazid, rifampicin, ethambutol and pyrazinamide) followed by 4 months continuation phase with isoniazid and rifampicin [17, 18]. For tuberculous meningitis and osteo-articular TB the continuous phase covered a period of 10 months [18, 19]. TB Treatment in children was directly observed with a care taker identified to be the DOTS provider. At the end of the continuation phase, treatment outcomes were declared as cured, treatment completed, treatment failed, died, lost to follow up and not evaluated defined in Table 1 [20]. The sum of children with outcomes “cured” and “treatment completed” made up those who were successfully treated.

**Table 1:** Definition of treatment outcomes

Outcome	Definition
Cured	A smear positive patient who completed treatment with a negative sputum smear in the final month of treatment and on at least one previous occasion
Treatment Completed	A sputum smear negative or extra-pulmonary TB registered patient or one with smear not done who has completed a full course of treatment
Treatment failed	A patient whose sputum smear is positive 5 months of treatment or later
Died	Patient who dies for any reason while on TB treatment
Lost to follow up	A patient with interrupted treatment for 2 or more consecutive months
Not evaluated	A person whose treatment outcome is unknown
Treatment success	A combination of cure and treatment completed

All children who were suspected to have TB were expected to be tested for HIV as per the National AIDS Control Program Guidelines (NACP) and those found to be positive initiated on ART within 2 weeks of starting TB treatment [21].

### Data collection and analyses

The data for the study was obtained from the database of patient information compiled from the 11 facilities participating in the case finding initiative over the period June 2010 to December 2013. This case finding initiative database was selected because it was readily available and included data on children less than 15 years, the age group of interest for the study.

The criteria for inclusion into the study sample consisted of all children less than 15 years diagnosed with smear positive and smear negative pulmonary TB, extrapulmonary TB and clinically diagnosed TB. Patients 15 years and older were excluded from the study population. The data were analysed using STATA 12. Descriptive analyses were conducted for demographic and clinical variables including gender, age, TB type, HIV status, facility type and year of TB registration. Those diagnosed with sputum smear negative TB and those clinically diagnosed with TB were combined into one group labelled clinically diagnosed pulmonary TB. The association between the independent variables and treatment outcomes were assessed using Chi-square test and Fisher’s exact test as relevant.

By way of definition, those designated as having “treatment success” consisted of patients who were cured and completed treatment while all other patients; those who died, were lost to follow up or failed treatment were classified as having poor treatment outcome. Odds ratio with 95% confidence interval (95%CI) were assessed from univariable (unadjusted) and multivariable (adjusted) logistic regression conducted to identify risk factors for mortality compared to those treated successfully. Given the relatively few variables being assessed, all variables were included in the preliminary multivariable regression model. A backward stepwise elimination process was then employed to account for confounders in the dataset. Significant variables were retained at a *p*-value of 0.05.

## Results

Over the course of the TB-case finding initiative, 219 children less than 15 years were diagnosed with TB representing 5.9% out of the total 3704 TB cases reported. The female:male ratio was 1:1.1. The median age was 3 years with a range of 3 months to 14 years and children less than 5 years made up 56.2% of the patients. Table 2 shows the characteristics of the study population.

**Table 2:** Demographic and clinical characteristics of children with TB in Accra, June 2010 to December 2013

Characteristic	Number (%)
Sex	
Male	113 (52.6)
Female	102 (47.4)
Age years	
<1	31 (14.4)
1–4	88 (40.9)
5–9	50 (23.3)
10–14	46 (21.4)
TB Type	
Clinically diagnosed pulmonary TB	79 (36.4)
Extra-pulmonary	37 (17.1)
Smear positive PTB	101 (46.5)
HIV status	
Negative	117 (54.7)
Positive	97 (45.3)
Facility	
Children’s Hospital	102 (46.6)
General Hospitals	66 (30.1)
Specialized Hospitals	36 (16.4)
Polyclinics	15 (6.9)

The majority of the children were new TB cases with the exception of 8 (3.8%) 7 of whom were retreatment cases and 1 relapse. Among the smear positive TB cases, 67 (66.3%) were aged less than 5 years and 79 (78.2%) were diagnosed in the children’s hospitals. Chest X-ray results were known for 162 (74%) of the children out of whom 159 (98%) were reported to have findings suggestive of TB.

**Fig. 1:**
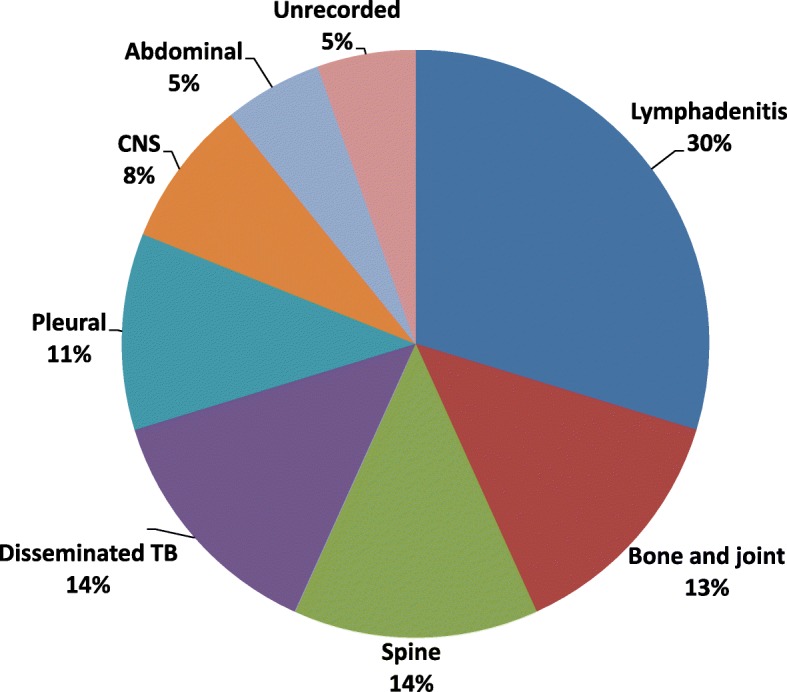
Distribution of extra-pulmonary infection sites among 37 children < 15 years in Accra, June 2010 to December 2013

A total of 79 children had clinically diagnosed pulmonary TB. Forty-five of them had a sputum smear negative result. Out of the remaining 34 children who did not have sputum microscopy done, 27 (80%) were less than 5 years and 29 (85%) were diagnosed in general hospitals. Among the 37 children (17%) with extra-pulmonary TB, lymph nodes were most commonly affected as shown in Fig. 1. Among the 5 children with EPTB in the bones and joints, the sites affected were femur (2), hip (2) and tibia (1).

**Fig. 2:**
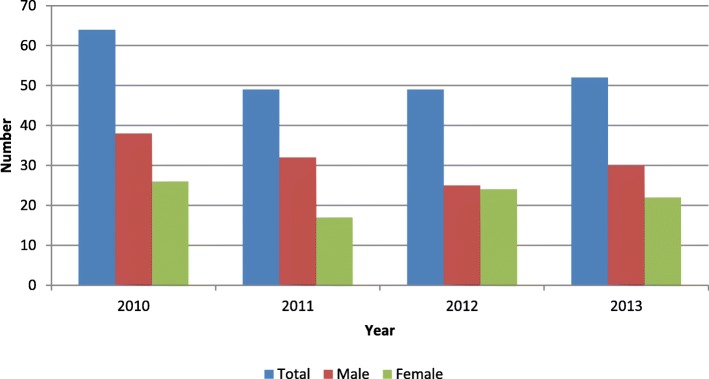
Distribution of children diagnosed with TB during TB case finding initiative by year and sex in Accra, June 2010 to December 2013

The HIV status was documented for virtually all the children (97.7%). Forty-four percent of the children were HIV positive. More children (30%) were diagnosed in 2010 from June to December compared to the other years which were full calendar years as shown in Fig. 2.

Almost half of the children (45.6%) were diagnosed in the children’s hospital. Less than 10% were diagnosed in the polyclinics. Of the 36 children diagnosed in the specialized hospitals, 4 came from the teaching hospital and the rest were from the regional hospital. There was no significant association between sex and the demographic and clinical variables analyzed.

### Treatment outcome

Treatment outcome was documented for 214 children (97.7%). One hundred and ninety-four of these, 90.7%, were successfully treated consisting of 81.3% who completed treatment and 9.4% who were cured. All the children were successfully treated in 2011. An adverse outcome was recorded for 20 patients; 18 (8.4% of the total with treatment outcome stated) died; 1 was not evaluated and 1 failed treatment. As shown in Table 3, there was significant association between treatment outcome and age, TB type, HIV status, facility attended.

**Table 3:** Treatment outcome of children with TB in Accra, June 2010 to December 2013, presented according to demographic and clinical variables

Characteristic	Cured Number (%)	Treatment completed Number (%)	Treatment failed Number (%)	Died Number (%)	Lost to follow up Number (%)	Not evaluated Number (%)	*P*-value
Sex							
Male	8 (7.2)	94 (84.7)	1 (0.9)	8 (7.2)	0	0	0.426
Female	11 (11.1)	77 (77.8)	0	10 (10.1)	0	1 (1.0)	
Age (years)							
<1	0	27 (93.1)	0	2 (6.90)	0	0	<0.001
1–4	2 (2.2)	76 (84.4)	0	12 (13.3)	0	0	
5–9	2 (4.0)	46 (92.0)	0	1 (2.00)	0	1 (2.0)	
10–14	16 (35.6)	25 (55.5)	1 (2.2)	3 (6.7)	0	0	
TB type							
Clinically diagnosed pulmonary TB	0	74 (96.10)	0	3 (3.90)	0	0	<0.001
EPTB	0	33 (91.67)	0	2 (5.56)	0	1 (2.78)	
Smear positive PTB	20 (20.20)	66 (66.67)	0	12 (12.12)	0	0	
HIV status							
Negative	18 (15.6)	90 (78.3)	0	6 (5.2)	0	1 (0.9)	0.001
Positive	1 (1.0)	81 (85.3)	1 (1.0)	12 (12.6)	0	0	
Unknown	1 (25.0)	3 (75.0)	0	0	0	0	
Facility							
Children’s Hospital	5 (5.0)	83 (83.0)	0	12 (12.0)	0	0	0.009
General Hospitals	9 (13.9)	53 (81.5)	0	3 (4.6)	0	0	
Specialized Hospitals	2 (5.7)	30 (85.7)	1 (2.9)	2 (5.7)	0	0	
Polyclinics	4 (28.6)	8 (57.1)	0	1 (7.1)	0	1 (7.1)	

The 10–14 year age group and the polyclinics had the highest proportion of those cured, 35.6 and 28.6% respectively. Treatment completion was highest among children with clinically diagnosed pulmonary TB (91.6%). The highest percentage of deaths was recorded among those in the age group 1 to 4 years (13.3%), HIV positive (12.6%), smear positive pulmonary TB (12%) and at the children’s hospital (12%). A male HIV positive child who had new smear positive PTB failed treatment while a female child with EPTB was not evaluated. No child was reported to have been lost to follow up.

The results of the regression analyses to assess predictors for mortality are shown in Table 4. Smear positive TB was identified as a risk factor for mortality in the univariable regression analyses. The preliminary multivariable logistic regression model showed that children with sputum smear positive TB (SPPTB) had higher odds of mortality compared to those with clinically diagnosed pulmonary TB [AOR = 6.1 (1.04, 35.57), *p* = 0.045]; and HIV positive children had a higher odds of mortality compared to those who were HIV negative [AOR = 3.85 (1.24, 11.4), *p* = 0.020]. In the final regression model, SPPTB [AOR = 4.21 (1.13, 15.6), *p* = 0.032] and HIV positivity [AOR = 3.24(1.15, 9.14), *p* = 0.026] remained risk factors for mortality.

**Table 4:** Univariable and multivariable logistic regression analyses showing the risk factors for mortality among children with TB in Accra, June 2010 to December 2013

Characteristic	Died	Successfully treated	Univariable analyses		Multivariable analyses	
	N (%)	N (%)	Unadjusted Odds Ratio (95% CI)	*p*-value	Adjusted Odds Ratio (95% CI)	*p*-value
Sex						
Male	8 (7.2)	103 (82.7)	Ref		Ref	
Female	10 (10.2)	88 (89.8)	1.42 (0.56, 3.58)	0.461	2.0 (0.71, 5.60)	0.187
Age						
< 1	2 (6.90)	27 (93.1)	Ref		Ref	
1–4	12 (13.3)	78 (86.7)	2.1 (0.44, 9.88)	0.358	2.1 (0.40, 10.9)	0.386
5–9	1 (2.0)	48 (98.0)	0.56 (0.07, 4.22)	0.576	0.74 (0.09, 6.11)	0.778
10–14	3 (6.8)	41 (93.2)	1.32 (0.22, 7.70)	0.760	1.24 (0.16, 9.93)	0.838
TB type						
Clinically diagnosed pulmonary TB	3 (3.9)	74 (96.1)	Ref		Ref	
EPTB	2 (5.7)	33 (94.3)	2.24 (0.43, 11.70)	0.338	2.75 (0.45, 16.88)	0.274
Smear positive PTB	12 (12.2)	86 (87.8)	3.73 (1.02, 13.59)	0.046	6.10 (1.04, 35.57)	0.045
HIV status						
Negative	6 (5.3)	108 (94.7)	Ref		Ref	
Positive	12 (12.8)	82 (87.2)	2.45 (0.93, 6.41)	0.069	3.85 (1.24, 11.4)	0.020
Facility						
Children’s Hospital	12 (12.0)	88 (88.0)	Ref		Ref	
General Hospitals	3 (4.6)	62 (95.4)	0.35 (0.10, 1.31)	0.120	1.30(0.21, 8.20)	0.778
Specialized Hospitals	2 (5.9)	32 (94.1)	1.22 (0.24, 6.14)	0.807	4.00 (0.45, 35.34)	0.212
Polyclinics	1 (7.7)	12 (92.3)	0.69 (0.18, 2.59)	0.580	1.85 (0.29, 11.7)	0.514

## Discussion

Calls for urgent action to protect children from TB and preventable deaths from this disease brings to the fore the increasing attention being paid to the global epidemic of childhood TB [22, 23]. This highlights the need to understand the characteristics and management outcomes of pediatric TB in various settings to inform appropriate planning and use of resources to enhance diagnosis, treatment and reporting in childhood TB services for better results [14]. In this study, the first to characterize the demographic, clinical and treatment outcomes of children diagnosed with TB in Accra, Ghana, children less than 15 years constituted about 6% of the total TB burden in the participating health facilities. Nine out of ten of the children had a successful treatment outcome. HIV positivity was a risk factor for mortality.

The proportion of childhood TB cases among the total TB cases recorded in this study is similar to figures reported for Ghana [24], and comparable to findings from studies in cities like Lagos (6.3%) and Abidjan (6.6%) which are also in the West African sub-Region [8, 9]. The global TB reports indicate that children less than 15 accounts for 7% of TB cases notified. [1, 25]. Assessing the true burden of childhood TB is fraught with a number of challenges including constraints in bacteriologically confirming TB in children, under-reporting and inadequate TB diagnostic capacity in facilities where children are seen. This is highlighted in our study which showed that most of the children clinically diagnosed with TB were from the general hospitals which may have had limited capacity to adequately investigate and confirm TB in children. At the time of the study smear microscopy was the main stay of TB diagnosis as molecular tests for TB had not yet become widely available. It is expected that with the subsequent Ghana NTP policy to roll out additional screening tools such as digital Chest X-ray and Gene Xpert equipment for testing coupled with appropriate training for health staff, early detection and reliable diagnosis of TB in children will improve [5, 24].

Several studies on childhood TB like ours report slightly more males than females with TB; in sync with the ratio of 1.1:1 reported globally [1, 6, 7, 9, 26–28]. Age wise, more than half of the TB cases in our study were in the younger age group. This is not unusual as younger children are reported to have a higher risk of progression from TB infection to disease [4, 29]. More than two out of five of the children in our study, were diagnosed with sputum smear positive TB similar to a study by Dangisso and colleagues in southern Ethiopia [27]. In contrast, other studies report higher proportions of sputum smear negative TB among children [8, 11, 30]. With TB in children being usually paucibacillary in nature and with the smaller amounts of sputum produced by children being swallowed, bacterial confirmation of TB in children is less common [31, 32]. It is possible that the high percentage of sputum smear positivity in our study may have been due to under diagnosis of smear negative and clinically diagnosed TB especially among younger children [27, 33]. Kunkel et al. argue that since children 4 years and below are less likely to be sputum smear positive (0.5%) than older children (14%) and adults (52%), reliance on sputum smear for diagnosis (which was the practice at the time of the case finding initiative) was associated with the risk of under diagnosing and under estimating TB in children.

The proportion of 17% EPTB among the study population was within range reported from studies in Benin [34], Turkey [35] and Cote D’Ivoire [8] while the finding of lymph nodes being the most commonly affected extrapulmonary TB site was also consistent with other studies [6, 36–41]. It is however interesting to note that globally, the proportion of EPTB among children with TB spans a wide range from 6 to 72% depending on the country reporting [8, 9, 26, 28, 30, 40]. A myriad of reasons have been suggested for this observation ranging from under-reporting and missing of EPTB cases, challenges with diagnosis, [28] to over diagnosis of EPTB and HIV infection rates of the population being studied [26, 28, 40]. The diagnosis of TB in children has long been recognized as challenging necessitating the use of multiple strategies including chest X-ray as a screening tool, clinical criteria and more sensitive molecular diagnostic methods [5, 41]. It was good to note that X-ray was being utilized in the TB diagnostic process in our study as three out of four of the children had an X-ray done compared to the 61% recorded in a Congolese study [26].

Unlike other studies in which as much as 75% of the study participants had no HIV status documented, the rate of HIV testing with results known among our study population was quite high [8, 26, 28, 30]. HIV status was not documented in only about 2%. This is commendable and showed that the health facilities were complying with the policy to test and counsel all TB patients for HIV [20].

The HIV infection rate in our children was similar to a study in Uganda [33] but relatively higher than other studies in Togo and Nigeria [9, 10]. In some of these studies, only a proportion of children had HIV status documented highlighting the possibility that the HIV infection rates reported may not have been a true reflection of the actual picture. HIV prevalence among children with TB from different parts of the world ranges from 10 to 60% [42]. Reported HIV-TB co- infection rates reflect the prevailing national HIV infection rates and other associated factors such as extent of immune suppression [43, 44]. This again reiterates the importance of HIV testing and counselling for all children with TB and TB evaluation for children with symptoms suggestive of TB or history of contact with a person with infectious TB [42].

The treatment success of over 90% in our study is in sync with the trend of treatment success reported for the Greater Accra Region of Ghana where this study was conducted [45]. Our level of treatment success was however generally higher than figures reported from studies conducted in a number of countries Ethiopia (85%), Nigeria (77%), Malawi (77%) in the African Region though lower than the 98% reported from Cote D’Ivoire [6, 8, 9, 46]. In these studies with lower treatment success, the poor outcomes were for the most part due to the patients being lost to follow up or not being evaluated. The treatment success recorded in our study may reflect good case management and the adherence to the DOTS strategy implemented by the NTP in Greater Accra Region in particular and the country as a whole [24]. Amo-Adjei in his paper assessing measures put in place by the NTP in Ghana and the relationship with treatment outcome found that the reduction in TB associated stigma among community members and health workers, the use of treatment supporters and the enablers’ package contributed to improved TB treatment outcomes and contributed to a two-fold increase from 44% in 1997 to 87% in 2010 and fewer patients lost to follow up [12]. In our study, mortality accounted for most of the children with poor outcome with the percentage of deaths higher in children with sputum smear positive TB and those co-infected with HIV. The mortality rate of 8.4% was not too far off from the figures reported from Malawi (9.5%), Botswana (10.5%) and Tanzania (10.9%), countries with a relatively higher HIV prevalence than Ghana [6, 47, 48]. In the logistic regression HIV positivity was associated with mortality, a finding corroborated in other studies [49]. Some of the possible reasons include the presence of other comorbidities, poor adherence due to an increase burden of drugs to take and drug resistance in co-infected children [40]. Considering the risk of latent TB developing into disease in persons living with HIV and the contribution of TB to deaths among those co-infected, implementation of the Three I’s for HIV/TB: intensified case-finding of TB (ICF), isoniazid preventive therapy (IPT) and infection control for TB provides opportunity to reduce morbidity and mortality in those without symptoms and signs of TB [50].

A review of the health sector TB program in Ghana indicated that IPT for children was not practiced universally neither was contact tracing conducted routinely [45]. These highlighted missed opportunities to identify children at risk of TB to protect them from developing the disease; and facilitate early diagnosis and initiation of treatment for those already with TB to limit negative outcomes. Subsequently, the NTP included the systemic screening of children as part of contact investigation and the promotion of IPT in its strategic plan [24]. Further studies are needed to assess the progress and outcome of the implementation of these strategies and the effect of preventing TB especially among HIV positive children. It was observed that 2010 recorded more TB cases over the 7-month period compared to the other full calendar years. This may have been due to the staff in the facilities adhering more closely to the case finding protocols in the first year soon after being trained for the new initiative as well as possibly more regular supervision in the starting months of implementation.

This study has some limitations. In making use of secondary data for the analyses, it was not possible to verify the diagnosis of TB in the study population nor distinguish between those that were probable and possible TB cases among those that were clinically diagnosed. It was also not possible to assess the clinical status of the children who were successfully treated especially since the majority was reported to have completed treatment. Another limitation is that there were some missing data in the database. Finally with the study population consisting of children with TB in Accra the findings may not be generalizable to those in other parts of the country. Notwithstanding these limitations, to our knowledge this is the first study to throw light on the treatment outcome of children derived from different categories of health facilities diagnosed and treated for TB in Ghana.

## Conclusions

This study highlights the demographic, clinical characteristics and treatment outcomes of children with TB in Accra, Ghana. Strengths identified in the childhood TB management include the high testing and documentation of HIV status in these children and the high treatment success recorded which met the target of END TB Strategy treatment success indicator. This baseline data on childhood TB in Accra throws a challenge to the NTP to at best maintain these standards especially as it rolls out case finding and preventive strategies as well as more sensitive TB diagnostic methods as outlined in the health sector tuberculosis strategic plan. The study also showed that HIV positivity was associated with lower treatment success while death accounted for the majority of those with poor treatment outcome. As the NTP rolls out its interventions, paying close attention to children with smear positive pulmonary TB and those who are HIV positive may reduce mortality among them and improve treatment outcomes.

## Data Availability

The dataset supporting the conclusions of this article is available in the Harvard Dataverse repository, 10.7910/DVN/KSTTWY

## Abbreviations

AOR: Adjusted Odds ration
DOTS: Directly Observed Treatment Short Course
EPTB: extra-pulmonary TB
GXP: Gene Xpert
HIV: Human Immuno-deficiency Virus
NACP: National AIDS Control Program Guidelines
NTP: National Tuberculosis Control Program
OPD: Outpatient department
PLHIV: Persons living with HIV
PTB: Pulmonary tuberculosis
SPPTB: Sputum smear positive TB
TB: Tuberculosis
WHO: World Health Organization
